# Consenting to health record linkage: evidence from a multi-purpose longitudinal survey of a general population

**DOI:** 10.1186/1472-6963-12-52

**Published:** 2012-03-05

**Authors:** Gundi Knies, Jonathan Burton, Emanuela Sala

**Affiliations:** 1 ISER, University of Essex, Wivenhoe Park, Colchester, Essex CO4 3SQ, UK; 2 University of Milano Bicocca, Milan, Italy

## Abstract

**Background:**

The British Household Panel Survey (BHPS) is the first long-running UK longitudinal survey with a non-medical focus and a sample covering the whole age range to have asked for permission to link to a range of administrative health records. This study determines whether informed consent led to selection bias and reflects on the value of the BHPS linked with health records for epidemiological research.

**Methods:**

Multivariate logistical regression is used, with whether the respondent gave consent to data linkage or not as the dependent variable. Independent variables were entered as four blocks; (i) a set of standard demographics likely to be found in most health registration data, (ii) a broader set of socio-economic characteristics, (iii) a set of indicators of health conditions and (iv) information about the use of health services.

**Results:**

Participants aged 16-24, males and those living in England were more likely to consent. Consent is not biased with respect to socio-economic characteristics or health. Recent users of GP services are underrepresented among consenters.

**Conclusions:**

Whilst data could only be linked for a minority of BHPS participants, the BHPS offers a great range of information on people's life histories, their attitudes and behaviours making it an invaluable source for epidemiological research.

## Background

The British Household Panel Survey (BHPS) is a renowned general topic social survey covering areas such as demographics, household composition, employment, education, training, health, values, opinions and finances. The survey started in 1991 and the same individuals and their households have been followed over time.

The BHPS assists with understanding the long-term effects of social and economic change, as well as policy interventions designed to impact upon the general well-being of the UK population. It is widely used in a number of different disciplines, but use in health research has been limited by the lack of objective measures of health. The BHPS collects self-reports of utilisation of health services (General Practitioner and hospital services as well as a range of other health and welfare services such as a health visitor, meals on wheels, social workers, family planning clinic) and of health status which give a holistic summary of the health condition of an individual. However, self-reports and medical records do not always match-up [1-4]. Whilst medical records contain objective measurements and confirmed diagnoses, they exclude diseases for which the patient has not consulted and they do not have much background information on the individual patient (e.g., education, employment status, financial well-being etc.). As a result, wherever possible it is best to use both approaches, i.e., survey data augmented with administrative records.

It is an ethical requirement in the UK that survey participants give written informed consent to data linkage before certain information can be combined. Previous studies have shown that 70-90% of the survey population allow access to their health records [5-7]. Sociodemographic characteristics and health profiles have been found to be different for consenters and non-consenters, implying that results on health of linked datasets might be misleading. The nature of the relationship between the different respondent characteristics and the propensity to consent remains unclear as characteristics that are associated with higher consent in one study are negatively associated with consent in another [8]. This may partly be explained by differences in the survey design and study populations; consent is the result of a complex decision making process that is influenced not only by standard socio-economic characteristics of the respondent but also by respondents' attitudes to privacy and the salience of the data linkage request, by survey design features such as survey fidelity and the presence of others during the interview, and, last but not least, by the interviewer's task-specific experience [9].

Previous evidence on consenting bias is from epidemiological surveys that focus on particular health outcomes, and on experience from data linkage requests on birth cohort studies, which are run by medical research units and have a strong focus on health-related issues. Participation in these studies required formal written consent, and the linkage to administrative health data is requested in the context of a study in which health and development is the primary research focus. The request is likely to appear to the respondent as valid and legitimate. Very little is known on consenting to health record linkage on surveys of the general population which have a much wider scope and survey the whole age range. Participation in these studies is based on informal informed consent, and there is less focus on health and so the request for data linkage may appear more "out of the blue", signing consent forms may be perceived threatening and respondents may be less likely to see the validity of the request. Whilst this may be expected to affect the level of consent, it may also affect patterns of consent. Ultimately, this is an empirical question.

This paper is an attempt to expand the current knowledge and exploits the rich resource of the BHPS data about those individuals who gave or withheld their consent. In 2008, in the 18th annual survey of the BHPS, participants were asked for their informed consent to allow their survey data to be augmented with health records held by the National Health Services (NHS) and the Departments of Health. Patterns of consent are analysed and how many linked records may be available for innovative analyses of health outcomes are extrapolated.

## Methods

The British Household Panel Survey (BHPS) is an annual longitudinal household panel survey, managed at the Institute for Social and Economic Research (ISER) at the University of Essex. The survey started in 1991 with a nationally-representative stratified, clustered sample of 5500 households. Within each household, all those aged 16 and above were eligible for a full individual interview. At Wave 2, in 1992, all individuals were issued for re-interview. From Wave 2 onwards, new entrants to the household are eligible for interview. If a sample member changed addresses, they are followed (within the UK) and interviewed in their new address. Any adults in their new address are also eligible for interview. As a young person reaches the age of 16 they become eligible for a full adult interview. A baby born to a sample household becomes part of the sample. Thus, the sample is designed to be self-perpetuating; whilst some sample members die, new sample members are joining all the time. This longitudinal design offers great potential for those who wish to research the life-course. In 1999 additional booster samples were added to the BHPS in Scotland and Wales (1500 households each) to allow for analysis within and across England, Scotland and Wales. In 2001, a Northern Ireland booster sample was added (2000 households). Generally the household response rates over the life of the BHPS have been in the range of 85-90%, although the last two waves dipped below this slightly (84.7 and 84.2% at Waves 17 and18, respectively). Our analysis focuses on the 13454 adults who gave a full interview and were living in the 7596 households which were interviewed at Wave 18 of the BHPS. Most interviews (96.6%) were carried out between September and December 2008, with the remainder being completed in the early months of 2009.

Interviews are conducted face-to-face using Computer-Assisted Personal Interviewing (CAPI) with interviewers calling on participants in their homes. The individual questionnaire takes around 45 min to answer, and questions cover a broad subject range. There are question modules on demographics, education, training, employment, values and opinions, politics, the environment, finances, receipt of benefits, external transfers and household expenditure. The BHPS also collects a considerable amount of self-reports on health, including, for example, the General Health Questionnaire (GHQ) to measure mental health, whether participants have been admitted to hospital, or diagnosed with a cancer. In waves 9 and 14, the survey includes the 36-item Health Survey (SF-36).

Approximately one week before the start of Wave 18 of the BHPS, sample members were sent an advance letter to inform them that an interviewer would soon be calling on them to ask for an interview. Consent to health data linkage was asked for the first time at Wave 18 and an information leaflet detailing the plans to add administrative health data to the survey was also enclosed with the advance letter. The leaflet set out what information would be added, who will use the information, how long consent lasts and data security. Two health-based consents were sought; (1) link to health data which covered admissions or attendance to hospital (including dates, diagnoses, treatments, surgical procedures, waiting times), records of specific conditions such as cancer or diabetes, prescriptions, and (2) link to the NHS Central Register to gather health registration information (such as name of Health Authority, NHS number, cause and date of death). There was also information for sample members about how they could revoke their consent at any time and gave a free-post address and a free-phone telephone number so they could contact ISER to ask any questions. Participants had the opportunity to request further information or to opt out of the survey on receipt of the advance letter. All materials assisting the collection of informed consent are provided online with the BHPS documentation [10].

Consents for both health data linkages were collected as part of the individual questionnaires from adults (16+ years old) at the end of the interview. The procedure for asking for consent, along with the actual question wording and content of the information leaflet and the consent forms, had to be approved by the Medical Research Ethics Committee (MREC). The MREC aimed to ensure that the consent request was transparent, and that the respondents were fully informed about what they were consenting to.

During the interview the interviewer explained to the participant about the data linkages, gave the participant a permission form to sign and also answered any additional questions the participant may have had about the linkages. Signing the permission form indicated that the participant had read the information leaflet and had the opportunity to ask questions about the process. On the form, there was a box for the participant to tick to indicate that they gave consent to linkage for a particular stream of data (health data, NHS Central Register), differentiated by which authorities hold the information. Participants were thus able to give consent to one stream of linkage and withhold their consent to another. The participants then had to sign and date each form and gave them to the interviewer. Forms were not left with the respondents to sign and mail back after the interviewer visit. However, a copy of the signed form was left with the participant for their own records. As well as receiving the signed consent form, the interviewer also coded in the CAPI questionnaire whether or not the participant gave consent. Once the fieldwork for Wave 18 of the BHPS was over, the data and the signed consent forms were returned to ISER. The consent forms were then checked against the CAPI data, to ensure that there had been no errors and that a signed consent form exists for all participants expected to have one according to the data. Where the CAPI data indicated consent, but there was no form, the CAPI data was edited to indicate that the form was missing and this was treated as not having consent.

The dependent variable used in this research is whether or not the participant signed a consent form to allow 'Flagging or tracing on the NHS Central Registers' or 'Adding of Administrative health records'. The independent variables are grouped into four blocks and include not only population characteristics that may be readily available in all medical research, say from health registrations (variable set 1, see Additional file 1: Table S1), but also characteristics that are not typically collected in medical surveys (variable sets 2-4, see Additional file 1: Table S1). Variable set 3 includes all health-related information collected in the BHPS interview in Wave 18; information on body mass and past hospitalisations is included from previous waves of the BHPS since obesity and hospitalisations are of specific interest to analysts and policymakers (and this was mentioned in the information leaflet). We report bivariate associations and estimate multivariate logistic regression models, allowing for spurious correlations in the characteristics. Results are weighted using population weights for the UK in 2008. The weights used are provided in the BHPS and account for unequal selection probabilities of addresses, non-response at the household level and non-response of individuals within responding households [11]. The consent rates are calculated in the statistical data analysis programme Stata using the command *mean *[12]. Adjusted Wald tests are performed to test for statistically significant differences in group means.

Finally, we estimate how many linked health records may be available, based on achieved consent rates. The calculation assumes that all those who have reported any hospitalisation over the course of the past 18 years do actually have a record on the respective country's hospital episode database, and, based on experience with linking hospital episodes from England to survey data, that 90% of current year hospitalisations and 75% of earlier hospitalisations can be correctly identified as belonging to the consenter [13]. Indeed, respondents are likely to have more than one hospital episode per hospitalisation, and they may have been hospitalised repeatedly.

## Results

All 13 454 adults who were interviewed were asked for their consent to health data linkage, and 5362 (41%) consented. While participants could allow linkage of one stream of data but not another, in practice 99% of those who gave consent to link to health data also gave consent to link to the NHS Central Register.

Additional file 1: Table S1 reports the number of observations and estimated consent rates broken down by population characteristics. Consent is significantly higher among people who live in England (Mean: 42.4; F(1, 13 453) = 56.91, *p *< .001), participants aged 16-24 (M: 45.9; F(1, 13 453) = 10.43, *p *< .001), and among those who consider their ethnicity to be British/Irish White (M: 42.4; F(1, 13 453) = 33.31, *p *< .001). There is some indication that consent is associated with level of qualification. There is a significant difference in consent for people who have a higher degree, A-levels, a commercial qualification or no qualifications. Consent is also higher among participants not living in standard household types (such as households consisting of two or more unrelated adults, e.g., student households), and among participants whose income falls in the third quartile of the income distribution. Self-reported health is generally not associated with consent. There are two exceptions; diabetes and obesity are associated with higher consent. Consent is higher for participants who reported that they have used other (non-GP or hospital in-patient) health and welfare services in the previous 12 months. No other indicator of use of health services is associated with consent in the bivariate models.

When we consider all four sets of independent variables in a multivariate logistic regression model, the negative associations between living in Wales, Scotland or Northern Ireland compared to living in England persist (Additional file 2: Table S2). Moreover, those who consider their ethnicity British/Irish White have a 75% higher chance of consent. We find that the youngest group of participants (aged 16-24) in the BHPS have a 61% higher chance of giving consent than the oldest group (aged 60 or older). Males are slightly overrepresented among consenters (OR: 1.14, *p *< .05). Whilst having a higher degree is positively associated with consent (OR: 1.52, *p *< .001), household and socio-economic characteristics of the population generally are not associated with consent. Last but not least, out of the 20 subjective indicators of health only one is associated with consent in the multivariate models. We find that participants who report to have been diagnosed with diabetes (OR: 1.30, *p *< .05) are more likely to consent. The association between consenting and utilisation of health services is complex. While people who have recently used GP services are less likely to consent (OR: 0.84, *p *< .001), there is a positive association with use of other health and welfare services in the last 12 months (OR: 1.15, *p *< .05), and with hospitalisations in a previous wave of the survey, where at least one hospitalisation was related to childbirth (OR: 1.24, *p *< .05). Recent hospitalisations and other indicators of use of health and welfare services are not associated with consent.

Additional file 3: Table S3 reports the estimated sample sizes for linking to health records. Consent to link health records was obtained from 2860 adults in England, and from roughly 2500 adults in the other countries of the UK. Given these numbers, analysts may expect to find complete linked health and survey records for around 2100 adults. Whilst not the subject of this paper, adults who were 'responsible' for a child (aged 0-15) in the household, usually the mother, were also asked for their permission to link administrative health data held on the child(ren) to the survey responses. Around 1500 consents for adding children's health records were obtained. For 90% of them, complete birth-related information would be available.

## Discussion

This research is the first population-based longitudinal study assessing the consent patterns in a population spanning the whole age range and in the context of a survey that is not specifically focused on health. We find that consent is biased only with respect to sociodemographic characteristics but not with respect to health and socio-economic characteristics. This is in stark contrast to the medical studies which report strong biases on health [6,7,14,15]. Consent rates, however, are much lower than in medical studies [5-7].

Differences to previous results on consenting bias may partly be explained by the fact that the BHPS recruited participants from the general population and the data linkage request covered a whole range of health data. As a general topic survey, the BHPS does not have the health and development focus of other studies which have asked for consent, so the request to link to administrative data may have been perceived as a peripheral request, unconnected to the core purpose of the study and equally threatening to all. Moreover, data linkage was a new area for the BHPS and it may be that respondents and interviewers, many of whom have participated for up to 18 years, were wary about this innovation. During the time in which the consents were asked there were also a number of high-profile losses of personal data by the government which may have reduced confidence in the security of personal administrative data.

Currently only a minority of BHPS records can be linked to health records given relatively low consent rates, although this still gives more than 5300 adults who have consented to data linkage. While consent is not biased with respect to the socio-economic and demographic characteristics of the core BHPS sample, the research potential for health in minority ethnic groups is limited due to relatively low numbers in the sample. In line with previous research, the analysis shows that minority ethnic groups are less likely to consent [8,16-18]. Despite the lower consent rates in Scotland, Wales and Northern Ireland, the over-sample in these areas means that the BHPS would be a potentially valuable resource to analyse regional differences. Moreover, the value of the BHPS arises from having followed the same people over a very long period of time and observing their lifestyle and household context. Hospitalisations have been reported by 50% of the respondents to BHPS Wave 18; some utilisations of hospital services may not have been reported [3]. The study observes the respondents (in the context of their household) prior to the hospitalisation, and afterwards.

Since most study characteristics are not associated with consent, analysts do not have to worry too much about re-weighting the analysis to adjust for differential consenting in most cases. In a companion paper it was found, however, that many other characteristics of respondents such as markers of their risk aversion and community-mindedness are associated with consent [9]. To the extent that these characteristics may also be related to health outcomes, analysts may consider more carefully whether re-weighting is necessary.

It is planned to re-approach non-consenters and new entrants to the study with the consent question in a future interview, and knowing who is more reluctant to consent may help targeting resources on groups of the population that currently are underrepresented among the consenters. The study results on diabetes and cancer, the only two types of disease which were specifically mentioned in the BHPS information leaflet, suggest that consent rates may be boosted by mentioning in the information leaflet specific diseases which have a high prevalence in the study population. The result is in line with findings from the survey research showing that co-operation rates are higher if the study subject is more salient to the participant [19].

## Conclusions

There is an interest in the UK in linking survey data to hospital episode records and, in the longer run, to make use of Primary Care Trust and GP data. The results of this study suggest that it may be difficult to obtain consent for such data linkages from recent users of GP services. Therefore, one may want to reconsider plans to link to GP records, i.e., remove any references in the information leaflet to types of health data which people may associate with their GP (here: prescriptions). In the UK it is not currently possible to systematically link surveys to GP records. Institutions that manage surveys may be best advised to design more specific consent forms and survey instruments when such linkages have become feasible.

In 2010, the BHPS sample was incorporated into *Understanding Society*, the new UK Household Longitudinal Study (UKHLS). For more information on this study see http://www.understandingsociety.org.uk. Future data linkage exercises on the survey will be informed by methodological research carried out on the Innovation Panel of *Understanding Society*. The Innovation Panel offers a unique opportunity to investigate the effect of different designs of survey instruments on survey outcomes, including informed consent to health data linkages.

## Competing interests

The authors declare that they have no competing interests.

## Authors' contributions

GK carried out the main analyses of the data. GK, JB and ES all contributed to the review of the literature, methods and discussion sections. All authors read and approved the final manuscript.

## Pre-publication history

The pre-publication history for this paper can be accessed here:

http://www.biomedcentral.com/1472-6963/12/52/prepub

## Supplementary Material

Additional file 1 **Table S1**. Characteristics of consenters in study sample. Click here for file

Additional file 2 **Table S2**. Multivariate logistic regression of consent to link administrative health records. Click here for file

Additional file 3 **Table S3**. Expected sample sizes for linked BHPS and health data. Click here for file
